# Protective Effect of Flavonoids from a Deep-Sea-Derived *Arthrinium* sp. against ox-LDL-Induced Oxidative Injury through Activating the AKT/Nrf2/HO-1 Pathway in Vascular Endothelial Cells

**DOI:** 10.3390/md19120712

**Published:** 2021-12-18

**Authors:** Jia-Rong Hou, Yan-Hong Wang, Ying-Nan Zhong, Tong-Tong Che, Yang Hu, Jie Bao, Ning Meng

**Affiliations:** Department of Biotechnology, School of Biological Science and Technology, University of Jinan, Jinan 250022, China; jiaronghou@163.com (J.-R.H.); wangyanhong1203@163.com (Y.-H.W.); zhongyingnan98@163.com (Y.-N.Z.); mls_chett@ujn.edu.cn (T.-T.C.); huyang922@163.com (Y.H.)

**Keywords:** vascular endothelial cell, flavonoid, oxidized low-density lipoprotein, Nrf2, apoptosis

## Abstract

Oxidized low-density lipoprotein (ox-LDL)-induced oxidative injury in vascular endothelial cells is crucial for the progression of cardiovascular diseases, including atherosclerosis. Several flavonoids have been shown cardiovascular protective effects. Recently, our research group confirmed that the novel flavonoids isolated from the deep-sea-derived fungus *Arthrinium* sp., 2,3,4,6,8-pentahydroxy-1-methylxanthone (compound **1**) and arthone C (compound **2**) effectively scavenged ROS in vitro. In this study, we further investigated whether these compounds could protect against ox-LDL-induced oxidative injury in endothelial cells and the underlying mechanisms. Our results showed that compounds **1** and **2** inhibited ox-LDL-induced apoptosis and adhesion factors expression in human umbilical vein vascular endothelial cells (HUVECs). Mechanistic studies showed that these compounds significantly inhibited the ROS level increase and the NF-κB nuclear translocation induced by ox-LDL. Moreover, compounds **1** and **2** activated the Nrf2 to transfer into nuclei and increased the expression of its downstream antioxidant gene HO-1 by inducing the phosphorylation of AKT in HUVECs. Importantly, the AKT inhibitor MK-2206 2HCl or knockdown of Nrf2 by RNA interference attenuated the inhibition effects of these compounds on ox-LDL-induced apoptosis in HUVECs. Meanwhile, knockdown of Nrf2 abolished the effects of the compounds on ox-LDL-induced ROS level increase and the translocation of NF-κB to nuclei. Collectively, the data showed that compounds **1** and **2** protected endothelial cells against ox-LDL-induced oxidative stress through activating the AKT/Nrf2/HO-1 pathway. Our study provides new strategies for the design of lead compounds for related cardiovascular diseases treatment.

## 1. Introduction

Atherosclerotic (AS) is a multifactorial inflammatory disease characterized by lipid deposition in the vessel wall, which seriously threatens human health [1,2]. Studies have shown that vascular endothelial injury is a key event in the occurrence and development of atherosclerosis [3,4]. Endothelial injury is characterized by vascular endothelial cell death, accompanied by a vascular inflammatory reaction, vascular wall morphological changes, and eventually triggers cardiovascular events [5]. Moreover, some factors are related to vascular endothelial cell injury, and oxidative stress is an important reason for atherosclerosis. It is reported that reactive oxygen species (ROS) can cause vascular endothelial cell dysfunction and damage to vascular wall cells [6]. As a main risk factor of atherosclerosis, hypercholesterolemia triggers the deposition of oxidized low-density lipoprotein (ox-LDL) under the intima in vascular walls [7,8]. Moreover, ox-LDL induces reactive oxygen species (ROS) overproduction, adhesion molecule release, and endothelial cell injury [9,10,11]. Therefore, protecting endothelial cells against ox-LDL-induced oxidative stress may be an effective strategy for the prevention and treatment of atherosclerosis. Drugs that preserve endothelial cells’ function during oxidative stress are urgently needed.

Marine fungi are a great natural source of lead compounds in drug discovery as they are rich in a great diversity of chemical molecules with unique structural features that exhibit various types of biological activities [12,13,14,15]. Flavonoids are well-known antioxidants and have been used as dietary supplements [16]. Recently, our research group isolated flavonoids from the deep-sea-derived fungus *Arthrinium* sp. UJNMF0008 and confirmed that 2,3,4,6,8-pentahydroxy-1-methylxanthone (compound **1**) and arthone C (compound **2**) had antioxidant activity in vitro [17]. Compounds **1** and **2** belong to xanthone compounds of flavonoids, and compound **2** was a new compound reported firstly by our research group [17]. It is well known that the xanthone compounds, mainly isolated from plants, microorganisms, lichens, and synthesis, have extensive biological activities, including antioxidants. For example, Johanis Wairata et al. isolated four xanthone compounds with strong antioxidant activity from Garcinia forbesii [18]. Tran et al. isolated Garcinoxanthone U, garcinone E, and 1,3,6,7-tetrahydroxyxanthone with DPPH scavenging activity from Garcinia mangostana [19]. Moreover, recent studies have shown that marine and terrestrial endophytic fungi are also an important mine of xanthone compounds, and more than 90 xanthone compounds have been reported from marine fungi [20]. For example, Wu et al. isolated 12 xanthones with ABTS scavenging activity from the metabolites of the deep-sea fungus *Aspergillus versicolor*. Importantly, it is reported that synthetic 1,3,5,6-tetrahydroxyxanthone protected endothelial cells from ox-LDL-induced adhesion of monocytes to endothelial cells [21]. Demethylbellidifolin, a major component of Swertia davidi, inhibited the adhesion of monocytes to endothelial cells by reducing the levels of tumor necrosis factor-α and endogenous nitric oxide synthase inhibitors [22]. Additionally, mangiferin has been shown to alleviate ox-LDL-induced VEC dysfunction [23]. These researches suggested that xanthone compounds may serve as protective agents for vascular endothelial cells. However, few marine-derived xanthones have been studied in vascular endothelial cells. In the present study, we further investigated whether compounds **1** and **2** could protect against ox-LDL-induced vascular endothelial cell injury and explored its potential actions on oxidative signaling events.

## 2. Results

### 2.1. The Compounds ***1*** and ***2*** Inhibited ox-LDL-Induced HUVEC Apoptosis and Adhesion Factors Expression

**Figure 1:**
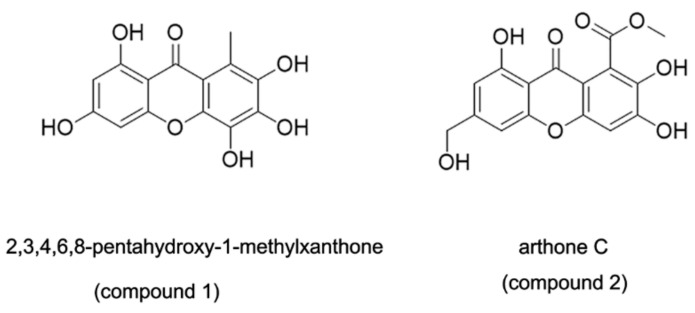
Chemical structures of compounds **1** and **2**.

**Figure 2:**
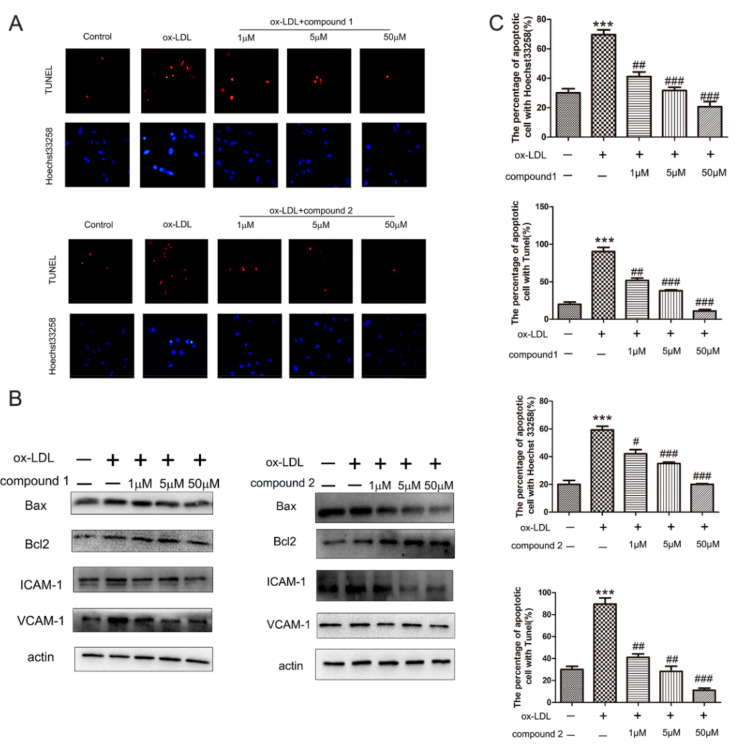
Effects of compounds **1** and **2** on ox-LDL-induced apoptosis and adhesion factors expression in HUVECs. HUVECs were treated with 50 μg/mL ox-LDL, 50 μg/mL ox-LDL and 1–50 μM compound **1** or **2** for 6 h (**A**) Nuclear fragmentation of cells by Hoechst 33258 and TUNEL staining (10×). (**B**) Western blot analysis of Bax, Bcl-2, VCAM-1, and ICAM-1 protein levels. (**C**) Bar chart showing quantification of HUVEC apoptosis percentage according to Hoechst 33258 and TUNEL staining. *** *p* < 0.001 vs. Control, ^#^ *p* < 0.05, ^##^ *p* < 0.01, ^###^ *p* < 0.001 vs. ox-LDL, *n* = 3.

Chemical structures of compounds **1** and **2** are shown in Figure 1. To assess the effects of compounds on ox-LDL-induced injury, we pretreated HUVECs with compounds **1** and **2** at different concentrations and stimulated the cells with 50 μg/mL ox-LDL for 6 h. Then, hoechst33258 staining was performed to detect nuclear DNA condensation and fragmentation, characteristics of apoptosis. We found that in the ox-LDL-treated groups, about 60–80% cells with nuclear condensation and fragmentation, while after the compounds **1** and **2** treatment, the percentage of apoptosis cells significantly decreased to around 20–40%. Results from the TUNEL assay showed that compounds **1** and **2** significantly reduced the number of TUNEL-positive cells (Figure 2A,C). The results of the Hoechst 33258 staining assay and TUNEL staining assay revealed that compounds **1** and **2** have protective effects on ox-LDL-induced HUVEC apoptosis.

To further explore the anti-apoptosis effect of compounds **1** and **2**, the expression of apoptosis-related proteins was detected by Western blotting. As presented in Figure 2B, the level of pro-apoptotic protein Bax decreased significantly, while that of anti-apoptotic protein Bcl-2 increased significantly in HUVECs after treatment with compounds **1** and **2**. These results confirmed that the compounds **1** and **2** had protective effects on ox-LDL-induced HUVEC apoptosis.

Next, we determined whether compounds **1** and **2** could inhibit the expression of endothelial adhesion molecules. The results of Western blot demonstrated that the protein level of VCAM-1 and ICAM-1 decreased after treatment with the compounds **1** and **2**, confirming that the compounds **1** and **2** inhibited ox-LDL-induced adhesion molecules expression in VECs (Figure 2B).

### 2.2. The Compounds ***1*** and ***2*** Significantly Inhibited the Accumulation of ROS and the Activation of NF-κB in HUVECs

**Figure 3:**
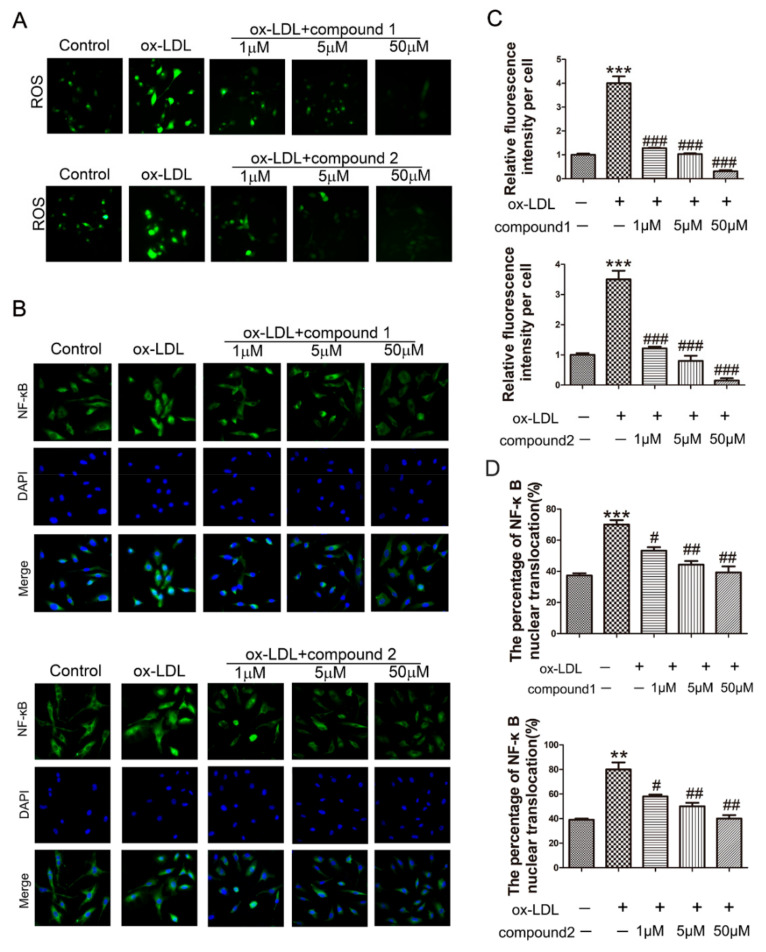
Compounds **1** and **2** inhibited the increased ROS level and NF-ĸB nuclear translocation induced by ox-LDL. HUVECs were treated with 50 μg/mL ox-LDL, 50 μg/mL ox-LDL and 1–50 μM compound **1** or **2** for 6 h. (**A**) The intracellular ROS level was examined by DCHF probe. (**B**) Fluorescent micrographs of NF-ĸB location in HUVECs. (**C**) The quantification of intracellular ROS level. (**D**) Bars showed the percentage of NF-ĸB nuclear translocation. ** *p* < 0.01, *** *p* < 0.001 vs. control, ^#^ *p* < 0.05, ^##^ *p* < 0.01, ^###^ *p* < 0.001 vs. ox-LDL, *n* = 3.

The effect of compounds **1** and **2** on ROS production in HUVECs was determined by using the DCFH-DA probe. Compared with the ox-LDL-treated group, the ROS levels were significantly reduced after the addition of compounds **1** and **2** (Figure 3A,C).

It is reported that the accumulation of ROS could promote NF-κB translocation into the nuclei and subsequently induce the expression of ICAM-1 and VCAM-1 [24]. Therefore, immunofluorescence staining was used to explore whether compounds **1** and **2** affect the location of NF-κB in ox-LDL-activated VECs. The results showed that NF-κB translocated into the nuclei after the cells were treated with ox-LDL, and this was dramatically inhibited by compounds **1** and **2** (Figure 3B,D).

### 2.3. The Compounds ***1*** and ***2*** Activated AKT/Nrf2/HO-1 Antioxidant Pathway

**Figure 4:**
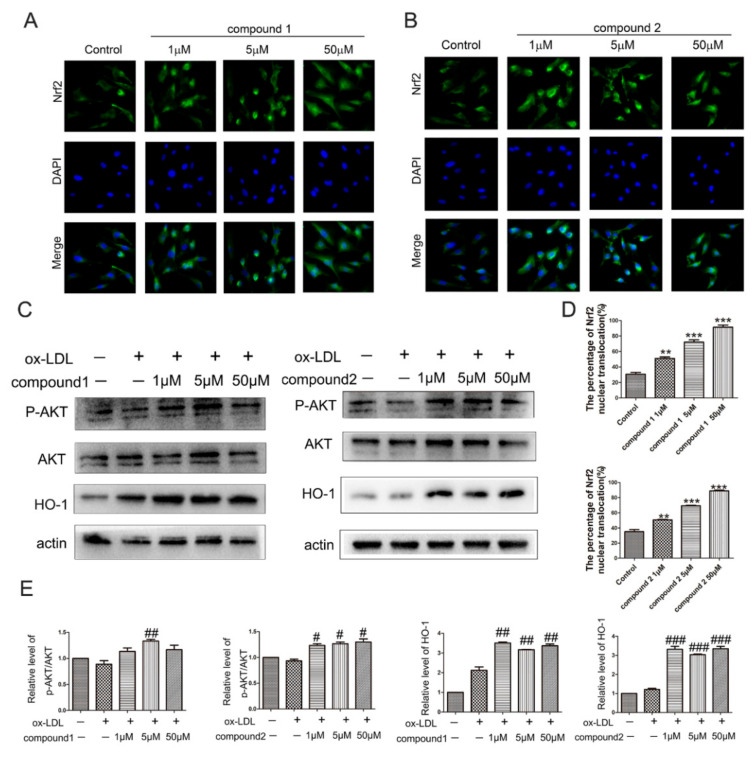
Effects of compounds **1** and **2** on AKT/Nrf2/HO-1 signaling pathway in HUVECs. (**A**,**B**) HUVECs were treated with 1–50 μM compound **1** or **2** for 6 h, fluorescent micrographs of Nrf2 location in HUVECs. (**C**) HUVECs were treated with 50 μg/mL ox-LDL, 50 μg/mL ox-LDL and 1–50 μM compound **1** or **2** for 6 h. The protein levels of phosphorylation AKT (pAKT) and HO-1 were examined by Western blot analysis. (**D**) Bars showed the percentage of Nrf2 nuclear translocation in (**A**,**B**). (**E**) Densitometry results of pAKT/AKT and HO-1 protein levels in panel (**C**). ** *p* < 0.01, *** *p* < 0.001 vs. Control, ^#^ *p* < 0.05, ^##^ *p* < 0.01, ^###^ *p* < 0.001 vs. ox-LDL, *n* = 3.

The transcriptional factor nuclear factor-(erythroid-derived 2)-like 2 factor (Nrf2) plays a major role in cellular antioxidant responses. It has been reported that cytoprotective gene heme oxygenase 1 (HO-1) is induced by Nrf2 nuclear translocation [25]. To elucidate the molecular mechanism of the protective effect of compounds **1** and **2** against ox-LDL-induced oxidative damage in HUVECs, we explored whether the compounds affect Nrf2/HO-1 pathway. By immunofluorescence staining, we found that the compounds **1** and **2** activated Nrf2 nuclear translocations in HUVECs (Figure 4A,B,D). Moreover, compared with the ox-LDL control group, the expression of HO-1 was significantly increased after the addition of compound **1** or **2** (Figure 4C,E).

In addition, it has been reported that AKT is the upstream gene of Nrf2. We further analyzed whether the compounds affect the phosphorylation of AKT. The results of Western blot demonstrated that the compounds **1** and **2** treatment increased the protein levels of pAKT (Figure 4C,E). Therefore, these results suggested that compounds **1** and **2** activated AKT/Nrf2/HO-1 antioxidant pathway.

### 2.4. Compounds ***1*** and ***2*** Protected VECs and Mediated Nrf2 Activation through AKT

**Figure 5:**
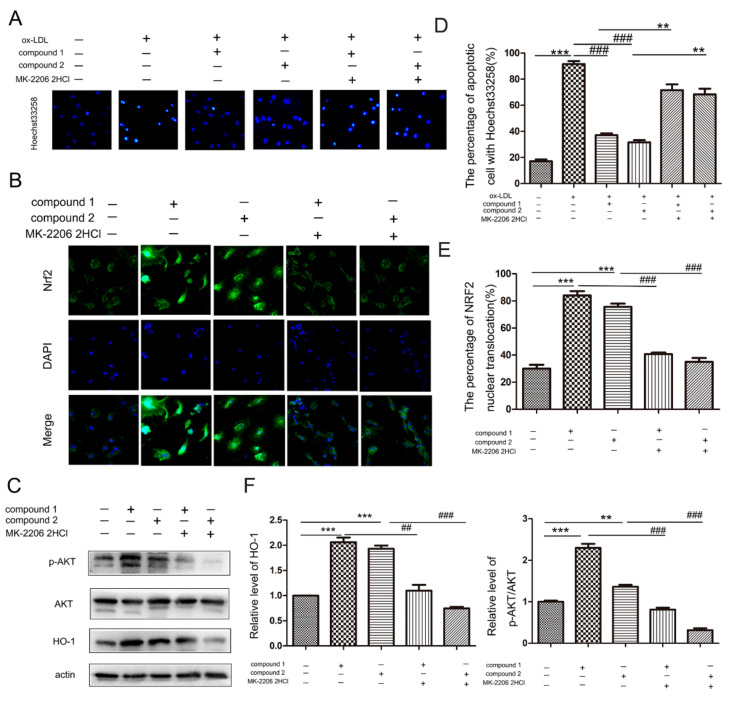
Inhibition of AKT abolished the effects of compounds **1** and **2** on ox-LDL-induced apoptosis and Nrf2/HO-1 activation. (**A**) HUVECs were pretreated with 5 μM MK-2206 2HCl for 1 h and treated with 50 μg/mL ox-LDL, 50 μg/mL ox-LDL and 50 μM compound **1** or **2** for 6 h. Nuclear fragmentation of cells by Hoechst 33258 staining. (**B**,**C**) HUVECs were pretreated with 5 μM MK-2206 2HCl for 1 h and treated with 50 μM compound **1** or **2** for 6 h. Fluorescent micrographs of Nrf2 location in HUVECs (**B**), Western blot analysis of HO-1 protein levels and phosphorylation levels of AKT (pAKT) (**C**). (**D**) Bar chart showing quantification of HUVEC apoptosis percentage according to Hoechst 33258 staining. (**E**) Quantitative analysis of Nrf2 nuclear translocation in panel (**B**). (**F**) Densitometry analysis of pAKT/AKT and HO-1 protein levels in panel (**C**). ** *p* < 0.01, *** *p* < 0.001, ^##^ *p* < 0.01, ^###^ *p* < 0.001 between the two values, *n* = 3.

Furthermore, MK-2206 2HCl (a specific inhibitor of AKT) was used to confirm whether the compounds protect VECs and mediate Nrf2 activation through AKT. The results of Hoechst33258 staining showed that MK-2206 2HCl blocked the anti-apoptosis effects of compounds in ox-LDL-stimulated HUVECs (Figure 5A,D). Importantly, compounds **1** and **2** neither activate Nrf2 into nuclei nor increase the HO-1 protein level in the presence of MK-2206 2HCl, suggesting these compounds activated Nrf2/HO-1 through AKT (Figure 5B,C,E,F).

### 2.5. Knockdown of Nrf2 Abolished the Inhibition Effects of Compounds on ox-LDL-Induced Apoptosis and Adhesion Factor Expression

**Figure 6:**
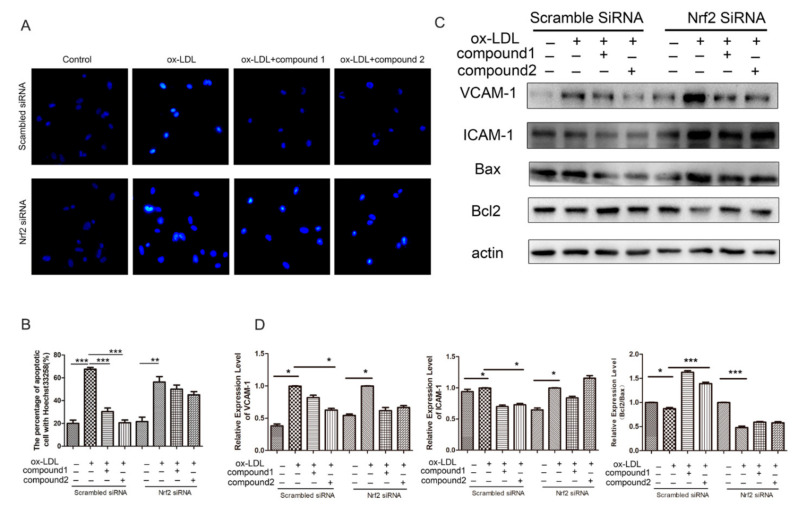
Compounds **1** and **2** protected against ox-LDL-induced injury via Nrf2 in HUVECs. HUVECs were treated with 40 nM Nrf2 siRNA or scramble siRNA for 48 h and stimulated with 50 μg/mL ox-LDL, 50 μg/mL ox-LDL, and 50 μM compound **1** or **2** up to 6 h. (**A**) Nuclear fragmentation of cells by Hoechst 33258 staining (10×). (**B**) Quantitative analysis of HUVEC apoptosis percentage according to Hoechst 33258 staining. (**C**) Western blot analysis of Bax, Bcl-2, VCAM-1, and ICAM-1 protein levels. (**D**) Densitometry analysis of VCAM-1, ICAM-1, and Bcl2/Bax protein levels in panel (**C**). * *p* < 0.05, ** *p* < 0.01, *** *p* < 0.001 between the two values, *n* = 3.

To further determine whether the protection of the compounds on VECs is Nrf2-dependent, we knocked down the Nrf2 expression using RNA interference. In cells treated with nonsilencing (scramble) siRNA, compounds **1** and **2** inhibited ox-LDL-induced VEC apoptosis, whereas, in Nrf2-knockdown cells, the effect of the compounds was suppressed (Figure 6A,B). Western blot analysis revealed that the effects of compounds **1** and **2** on ICAM-1, VCAM-1, BAX, and Bcl2 protein levels were inhibited by knockdown of Nrf2 (Figure 6C,D). Therefore, our results suggested that compounds protected endothelial cells from ox-LDL-induced injury via activating Nrf2 anti-oxidation pathway.

### 2.6. Knockdown of the Nrf2 Suppressed the Effects of the Compounds ***1*** and ***2*** on the Increased ROS Level and the NF-κB Nuclear Translocation Induced by ox-LDL in HUVECs

**Figure 7:**
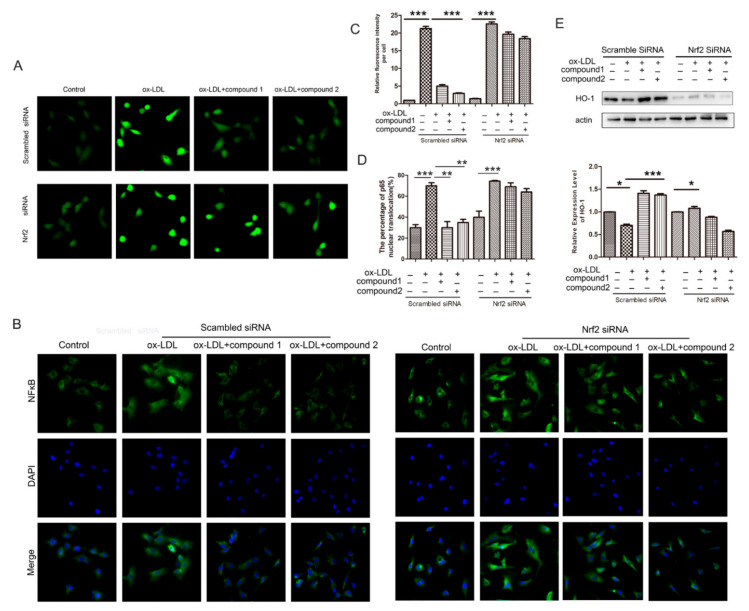
Knockdown of Nrf2 suppressed the inhibitory effects of compounds **1** and **2** on the accumulation of ROS and the activation of NF-κB induced by ox-LDL in HUVECs. HUVECs were treated with 40 nM Nrf2 siRNA or scramble siRNA for 48 h and stimulated with 50 μg/mL ox-LDL, 50 μg/mL ox-LDL, and 50 μM compound **1** or **2** up to 6 h. (**A**)The intracellular ROS level was examined by DCHF probe. (**B**) Fluorescent micrographs showed the location of NF-ĸB in HUVECs. (**C**) Quantitative analysis of intracellular ROS in panel (**A**). (**D**) Quantitative analysis of NF-ĸB nuclear translocation percentage in panel (**B**). (**E**) Western blot analysis of HO-1 protein level, bar graph showed the HO-1 protein level compared with β-actin. * *p* < 0.05, ** *p* < 0.01, *** *p* < 0.001, between the two values, *n* = 3.

Additionally, we examined the effects of the compounds on ROS level and NF-κB nuclear translocation in which Nrf2 was knocked down. The results showed that compounds **1** and **2** did not inhibit ox-LDL-induced increase in ROS level and NF-κB nuclear translocation when the VECs were knockdown of Nrf2, confirming these compounds inhibited ox-LDL-induced oxidative stress through Nrf2 (Figure 7).

## 3. Discussion

In this study, we demonstrated that xanthone compounds of flavonoids from deep-sea-derived *Arthrinium* sp. compounds **1** and **2** exhibited protective effects on ox-LDL-stimulated vascular endothelial cells via activating AKT/Nrf2/HO-1 anti-oxidative signal pathway.

Endothelial cell injury is believed to be the initial step in the atherosclerosis process [26]. A series of studies have indicated that ox-LDL, a main risk factor of atherosclerosis, contributes to endothelium injury by inducing endothelial cell oxidative stress [27]. Therefore, inhibition of endothelial cell oxidative injury by chemical small molecules is an effective strategy for the treatment of atherosclerosis. Recently, a series of new flavonoids were isolated from a marine-derived fungus *Arthrinium* sp. by our research group [17]. It is known that flavonoids are potentially involved in cardiovascular prevention, mainly by decreasing oxidative stress [28]. Our previous studies have shown that among these marine-derived flavonoids, compounds **1** and **2** exhibited potent antioxidant properties with DPPH and ABTS radical scavenging activities in vitro [17]. Compounds **1** and **2** belong to xanthone compounds of flavonoids. Interestingly, some research has demonstrated that xanthone compounds, such as synthetic 1,3,5,6-tetrahydroxyxanthone, demethylbellidifolin, and mangiferin exhibited protective effects in VECs [22,23,29]. However, marine-derived xanthones protect vascular endothelial cells from ox-LDL-induced injury toward atherosclerosis are limited. Here, we further explored the effects of compounds **1** and **2** on ox-LDL-stimulated HUVECs. The results revealed that compounds **1** and **2** significantly inhibited ox-LDL-induced apoptosis and the increase in ICMA-1 and VCAM-1 protein levels.

Through the DPPH scavenging activity test of 30 xanthone compounds by Xican Li, it is speculated that the structure of hydroquinone or catechol plays an important role in the antioxidant activity of these compounds, which is consistent with our previous reports that compounds **1** and **2** possess the structure of hydroquinone [30]. Moreover, it is reported that ROS accumulation induced by ox-LDL promotes NF-κB translocation into the nuclei and subsequently induces endothelial cell injury [31]. In this study, our results confirmed that compounds **1** and **2** significantly inhibited the accumulation of ROS and the activation of NF-κB in HUVECs, suggesting that these two compounds may protect endothelial cells through antioxidant pathways.

Nrf2 is an important transcription factor that regulates the cellular oxidative stress response. Nrf2 promotes the transcription of various downstream antioxidant genes, including hemeoxygenase-1 (HO-1) [32]. It has been shown that Nrf2 activators decreased the probability of developing atherosclerotic lesions by decreasing oxidative stress [33]. Moreover, some xanthone compounds exert cell-protective effects by activating Nrf2 [34]. For example, euxanthone activated Nrf2 and protected ox-LDL-induced endothelial cell injury [35]. Mangiferin inhibited doxorubicin-induced VEC apoptosis via the Nrf2/HO-1 signaling pathway [36]. Therefore, we detected whether compounds **1** and **2** protect VECs against ox-LDL-induced oxidative damage by affecting Nrf2/HO-1 pathway and found that these compounds activated Nrf2 to translocate into nuclei and increased HO-1 protein level. Importantly, knockdown of Nrf2 by siRNA inhibited the antioxidant and protective effects of the compounds on the endothelium. These results revealed that compounds **1** and **2** exhibited antioxidant stress and protective effects by activating Nrf2 in VECs. In addition, it has been reported that Nrf2 activation is regulated by AKT, and inhibition of Akt attenuates Nrf2 activation [37,38]. We found that compounds **1** and **2** increased the phosphorylation level of AKT. However, inhibition of AKT activity significantly inhibited Nrf2 activation and the protective effect of the compounds on vascular endothelial cells, indicating these compounds mediated Nrf2 antioxidant pathway through AKT.

**Figure 8:**
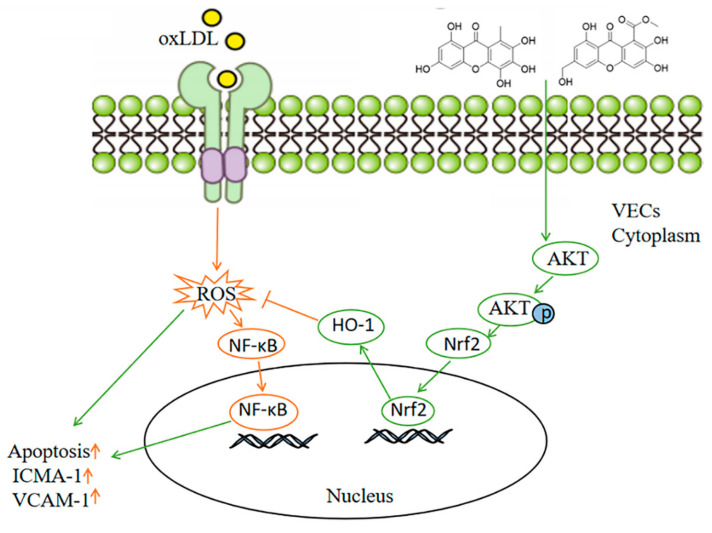
Scheme summarizing the mechanism for inhibition effects of compounds **1** and **2** on ox-LDL-induced VEC injury. Ox-LDL induces ROS generation and activates NF-ĸB nuclear translocation, which leads to VEC apoptosis and adhesion molecule expression. Compounds **1** and **2** activate Nrf2 nuclear translocation by inducing AKT phosphorylation. The nuclear Nrf2 facilitates the expression of HO-1, which inhibits cellular ROS production and NF-ĸB activation induced by ox-LDL.

In summary, our results revealed two novel flavonoids from deep-sea-derived fungus *Arthrinium* sp., which named compounds **1** and **2** significantly inhibited ox-LDL-induced VEC apoptosis and adhesion factors expression. Mechanism studies further elucidated that the compounds **1** and **2** activated AKT/Nrf2/HO-1 signaling pathways and further inhibited ox-LDL-induced ROS overproduction and NF-κB activation (Figure 8). Therefore, our study provides a possible therapeutic avenue for the treatment of atherosclerosis.

## 4. Materials and Methods

### 4.1. Reagents

Medium M199 and bovine serum were obtained from Gibco Co. (Carlsbad, CA, USA). Protein was extracted from cells using radio immunoprecipitation assay (RIPA) lysis buffer (Dingguo Changsheng Biotechnology Co., Ltd., Beijing, China). The primary antibodies anti-Bcl-2, anti-Bax, anti-HO-1, anti-ICAM-1, anti-VCAM-1, anti-phospho-AKT, and anti-AKT were acquired from Proteintech Group, Inc. (Chicago, IL, USA). The peroxidase-conjugated secondary antibodies were obtained from Dingguo Changsheng Biotechnology Co., Ltd. (Beijing, China).

### 4.2. Cell Culture

HUVECs were obtained from ScienCell (San Diego, CA, USA) and cultured on gelatin-coated plastic dishes in M199 medium with 20% (*v*/*v*) bovine serum and 10 IU/mL fibroblast growth factor 2. HUVECs were maintained at 37 °C under humidified conditions and 5% CO_2_. Morphologic changes of HUVECs were observed by inverted phase-contrast microscopy (ZEISS, Primovert, Germany).

### 4.3. Immunofluorescence Assay

After treatment, the culture medium was removed by washing with PBS. Then, HUVECs were fixed with 4% paraformaldehyde and blocked with PBS containing 3% goat serum for 20 min at room temperature. After blocking, cells were incubated with p65 or Nrf2 primary antibody (1/100) at 4 °C overnight. After three rinses in 0.1 M PBS, cells were treated with corresponding fluorescence-labeled secondary antibodies (1/200) in a humid chamber at 37 °C for 1 h. After washing with PBS, the immunofluorescence was examined by an inverted fluorescent microscope (Leica, Wiesbaden, Germany).

### 4.4. Western Blot Analysis

Protein was extracted from HUVECs using radio immunoprecipitation assay (RIPA) lysis buffer (Dingguo Changsheng Biotechnology Co., Ltd., Beijing, China). The protein concentration was determined using BCA Protein Assay kits (Beyotime, Shenzhen, Guangdong, China). Equal amounts of protein lysates (15 μg per lane) underwent 15% SDS-PAGE, and separated proteins were transferred to PVDF membranes (Millipore, Madison, WI, USA). The membranes were blocked at room temperature for 1.5 h in 5% non-fat dry milk diluted with TBST to reduce non-specific binding. The membranes were incubated overnight at 4 °C with primary antibodies. After washing with TBST three times, membranes were incubated with HRP-conjugated secondary antibody (Dingguo Changsheng Biotechnology Co., Ltd., Beijing, China) for 1.5 h at room temperature. Immune complexes were detected by enhanced chemiluminescence (Millipore Corporation, Billerica, MA, USA). Integrated densities of bands were quantified by Image J software.

### 4.5. TUNEL Staining

TUNEL staining was performed according to the manufacturer’s protocol using In Situ Cell Death Detection Kit, TMR red (Roche, Switzerland, France). DAPI staining reagent (Beyotime, China) was used to stain nuclei. Briefly, after all treatments, HUVECs were fixed with 1% buffered formaldehyde for 30 min and subsequently permeabilized with 0.25% Triton X-100 in 0.1% sodium citrate for five minutes. Then, the fixed cells were stained using fluorescein-conjugated TUNEL. The TUNEL-positive cells were observed under a fluorescence microscope (LEICA, Germany). Eight visual fields were selected from each group to calculate the proportion of TUNEL-positive cells.

### 4.6. Hoechst33258 Staining for Apoptosis

After treatment, HUVECs were washed twice with PBS. Then, HUVECs were stained with 10 mg/mL Hoechst 33258 (Dingguo Changsheng Biotechnology Co., Ltd., DH163) at 37 °C for 15 min and washed with PBS three times. At last, the cells were photographed under a fluorescence microscope (LEICA DMi8, Wiesbaden, Germany). The nuclear condensation and fragmentation of cells were used as indicators of apoptotic cells.

### 4.7. Intracellular ROS Assay

The levels of intracellular ROS were detected using the fluorescent probe 2′,7′-dichlorodihydrofluorescein (DCHF, Invitrogen, Carlsbad, CA, USA). After treatment, cells were incubated with DCFH-DA (10 mM) for 30 min at 37 °C in 5% CO_2_ and washed with PBS three times. The cellular fluorescence was monitored under a fluorescence microscope (LEICA DMi8, Wiesbaden, Germany). The amount of ROS was quantified as the relative fluorescence intensity of DCF per cell within the sight area.

### 4.8. Transient Transfection and RNA Interference

HUVECs at 60–70% confluence were transfected with Nrf2 or scrambled siRNA using a Lippo2000 transfection reagent according to the manufacturer’s protocol (Invitrogen, Waltham, MA, USA). After incubation for 6 h, the transfection medium was removed and replaced with fresh normal growth medium for 24–48 h.

### 4.9. Statistical Analysis

Data are presented as mean ± SEM. Images were processed by use of Graphpad Prism 5 (GraphPad Software, La Jolla, CA, USA) and Adobe Photoshop (Adobe, San Jose, CA, USA). At least three independent replications were performed. One-way ANOVA combined with Bonferroni post-hoc tests were used to determine the significance between different groups. *p* < 0.05 was considered statistically significant.

## Data Availability

The data presented in this study are publicly available.
